# Limitations of Ribotyping as Genotyping Method for *Corynebacterium ulcerans*

**DOI:** 10.3201/eid2610.200086

**Published:** 2020-10

**Authors:** Tsuyoshi Sekizuka, Chihiro Katsukawa, Makoto Kuroda, Keigo Shibayama, Ken Otsuji, Mitsumasa Saito, Akihiko Yamamoto, Masaaki Iwaki

**Affiliations:** National Institute of Infectious Diseases, Tokyo, Japan (T. Sekizuka, M. Kuroda, K. Shibyama, A. Yamamoto, M. Iwaki);; Osaka Prefectural Institute of Public Health, Osaka, Japan (C. Katsukawa);; University of Occupational and Environmental Health, Kitakyushu, Japan (K. Otsuji, M. Saito)

**Keywords:** Corynebacterium ulcerans, molecular typing, genome sequence, multilocus sequence typing, phylogeny, bacteria, zoonoses, ribotyping, whole-genome sequencing

## Abstract

We conducted molecular typing of a *Corynebacterium ulcerans* isolate from a woman who died in Japan in 2016. Genomic DNA modification might have affected the isolate’s ribotyping profile. Multilocus sequence typing results (sequence type 337) were more accurate. Whole-genome sequencing had greater ability to discriminate lineages at high resolution.

*Corynebacterium ulcerans* is a zoonotic pathogen that causes an illness categorized in World Health Organization documents as diphtheria (*1*). Genotyping methods such as ribotyping, multilocus sequence typing (MLST), and whole-genome sequencing are used to classify isolates. During the 1990s and early 2000s, the standard molecular typing method of *Corynebacterium diphtheriae* was conventional ribotyping (*2*,*3*). Ribotyping is also used to classify *C. ulcerans* (*4*) and compare isolates (*5*–*9*). Today, the standard method is MLST because of its objectivity and reproducibility (*8*,*10*). We sequenced 3 isolates of *C. ulcerans* from patients in Japan to analyze the accuracy of conventional ribotyping, MLST, and whole-genome sequencing.

## The Study

In 2016, a 66-year-old woman in Fukuoka, Japan, died of a diphtheria-like disease. Otsuji et al. isolated toxigenic *C. ulcerans* from the patient’s tracheal pseudomembrane and blood (*6*). We analyzed the isolate (FH2016-1) from the pseudomembrane alongside the first (*11*) and second (*5*) *C. ulcerans* isolates taken from patients in Japan; the first isolate (0102) was taken in 2001 and the second isolate (0211) in 2002.

**Figure 1:**
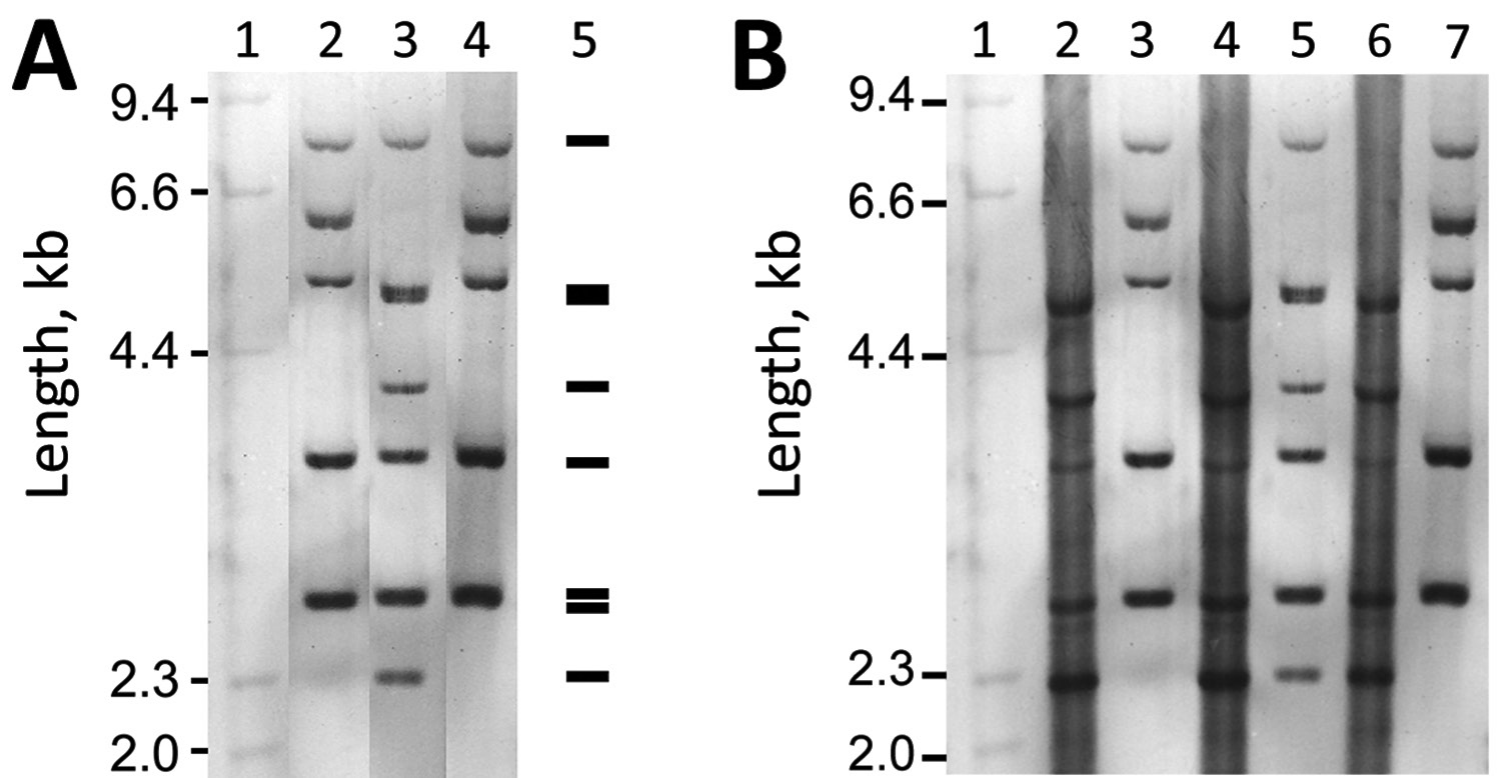
Alteration of ribotyping patterns by genomic DNA modification of *Corynebacterium ulcerans* strains 0102, 0211, and FH2016–1, Japan, 2001–2016. Ribotyping was performed as described previously (*4*,*11*). *Hin*dIII-digested, digoxigenin-labeled λ phage DNA segments were used as length markers. A) Conventional ribotyping patterns of strains 0102, 0211, and FH2016-1. 1, λ*Hin*dIII; 2, 0102; 3, 0211; 4, FH2016-1; 5, Pattern predicted by in silico typing. B) Ribotyping patterns of genomic DNA and whole-genome amplified DNA as substrates. 1, λ*Hin*dIII; 2, 0102 WGA; 3, 0102 native; 4, 0211 WGA; 5, 0211 native; 6, FH2016-1 WGA; 7, FH2016-1 native. The label “WGA” indicates whole-genome amplified DNA as a substrate; “native” indicates genomic DNA. WGA (unmodified) DNA of the 3 strains show identical patterns. The pattern matches that of native 0211 (unmodified genomic DNA). In contrast, native FH2016-1 and 0102 are modified and show different patterns from their WGA counterparts.

Strains 0102 and 0211 (named for the initial isolates taken in 2001 and 2002) are the 2 major ribotypes of *C. ulcerans* in Japan. Our conventional ribotyping of the isolates found the pattern obtained from FH2016-1 was indistinguishable from that of 0102, indicating that FH2016-1 belongs to strain 0102 (Figure 1, panel A).

We also whole-genome sequenced strains FH2016-1 and 0211 using the NextSeq500 Illumina (for strain FH2016-1 [Illumina, https://www.illumina.com]), Illumina GAII (for strain 0211 [Illumina]), ABI 3730xl (Thermo Fisher, https://www.thermofisher.com), and PacBio Sequel (Pacific Biosciences of California, Inc., https://www.pacb.com) sequencers, followed by de novo assembly. We deposited complete sequences and assembly methods in GenBank under accession nos. AP019663 (strain FH2016-1) and AP019662 (strain 0211). Using these sequences and the previously published genome sequence (*12*) of strain 0102 (GenBank accession no. AP012284), we conducted in silico ribotyping of *Bst*EII-digested fragments that hybridized with OligoMix5 probes, producing a predicted pattern for each sequence (*13*). The predicted patterns of all 3 strains matched the conventional ribotype pattern of strain 0211. However, the conventional ribotyping patterns of strains FH2016-1 and 0102 did not match the in silico–predicted ribotype pattern (Figure 1, panel A).

The discrepancy between the conventional and in silico–predicted patterns is caused by impaired restriction digestion at specific *Bst*EII sites. In these strains, the conventional (modified) ribotype pattern differed from the in silico–predicted (unmodified) ribotype pattern by a shift of 4 fragments (Appendix Figure 1, panel A). For example, in silico typing predicted that 3 *Bst*EII sites would be digested at nt 770,000 of strain FH2016-1. PacBio modification analysis revealed that 1 of these sites might have been modified (Appendix Figure 1, panel B). *Bst*EII is sensitive to methylation and other types of DNA modification (*14*). Thus, the difference in restriction fragment patterns was closely related to the nucleotide modifications within *Bst*EII recognition sites (Appendix Figure 1, panel B). Other *Bst*EII sites also might have been modified, resulting in the 4-fragment shift. Accordingly, we did not observe this shift in ribotypes of unmodified DNA substrate prepared by whole-genome amplification of the 3 strains (*15*) (Figure 1, panel B). The patterns of unmodified DNA matched the pattern of strain 0211 (Figure 1, panel B) and the in silico–predicted pattern (Figure 1, panel A). The >6.1-kb bands seen in “native” lanes were not visible in whole-genome amplification lanes, potentially because of the failure of whole-genome amplification to generate large fragments. These results indicate that ribotyping patterns might be substantially affected by DNA modification.

**Figure 2:**
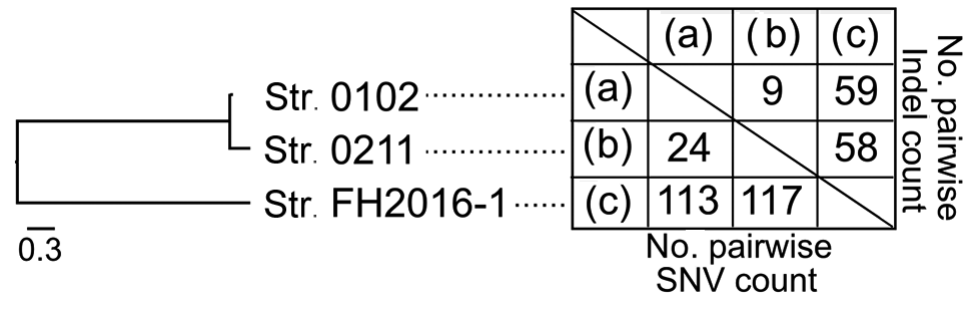
Genetic similarity among 3 selected strains of *Corynebacterium ulcerans*, Japan, 2001–2016. Strain 0102 is represented by (a), strain 0211 by (b), and strain FH2016–1 by (c). Numbers of SNVs and indels between strains are shown. A phylogenetic tree generated by SNV data are shown on the left. Indel, insertion/deletion; SNV, single-nucleotide variation.

The sequences of strains FH2016-1, 0102, and 0211 were highly homologous. For example, they shared complete sequence identity (data not shown) for a structural gene (locus tag CULCFH20161_03390) encoding a DNA methylase. However, we observed small differences in their genomes (Table; Figure 2; Appendix Table 1). We expected factors contributing to genomic DNA modification to be common between strains FH2016-1 and 0102, but not 0211. Scanning the genomes of the 3 strains for such factors resulted in 15 candidate open reading frames (ORFs) (Table). None of these ORFs contained motifs related to DNA methylation; however, these ORFs might still contribute to DNA modification of other gene products. The nature of the modification(s) remains unknown.

**Table:** Conserved mutation sites among strains 0102, 0211, and FH2016–1 of *Corynebacterium ulcerans*, Japan, 2001–2016*

Position (strain 0102)	Strain 0102	Strain 0211	FH2016–1	Mutation type	Locus_tag, gene	ORF length, bp	Product	Detected mutation in each ORF	Amino acid substitution	Description
13,243	T	TTC	T	Insertion	CULC0102_0011	705	Putative membrane protein	293T>TTC	NA	Pseudogene in strain 0211
147,936	T	C	T	SNV	CULC0102_0143	873	Putative ABC transporter, substrate binding protein	476T>C	Leu159Ser	NA
270,551	AT	A	AT	Deletion	CULC0102_0261	3,204	Putative surface-anchored membrane protein	751AT>A	NA	Pseudogene in strain 0211
408,576	C	T	C	SNV	CULC0102_0389 (menD)	1,614	2-succinyl-5-enolpyruvyl-6-hydroxy-3-cyclohexene-1-carboxylate synthase	1574C>T	Ala525Val	NA
561,350	A	AT	A	Insertion	CULC0102_0552	297	Hypothetical protein	218A>AT	NA	Pseudogene in strain 0211
820,419	G	T	G	SNV	CULC0102_0788	5,073	Putative helicase	2928G>T	Lys976Asn	GO term: DNA metabolic process
989,653	C	T	C	SNV	CULC0102_0951	1,143	Hypothetical protein	965G>A	Gly322Asp	NA
1,058,136	G	A	G	SNV	CULC0102_1010	468	Putative membrane protein	88G>A	Gly30Ser	NA
1,094,809	T	C	T	SNV	CULC0102_1045	1,143	Putative glutathione S-transferase	55A>G	Thr19Ala	NA
1,709,596	G	T	G	SNV	CULC0102_1586 (trpC2)	810	Indole-3-glycerol phosphate synthase	291C>A	His97Gln	NA
1,886,000	C	T	C	SNV	CULC0102_1749 (hrcA)	1,047	Heat-inducible transcription repressor	232G>A	Val78Met	GO term: DNA metabolic process
2,038,758	GGC...GCA (123 bp)	G	GGC...GCA (123 bp)	Deletion	Intergenic_region	NA	NA	NA	NA	NA
2,104,276	G	A	G	SNV	CULC0102_1940	393	Hypothetical protein	346C>T	Arg116Cys	NA
2,126,945	C	T	C	SNV	CULC0102_1961	1,185	Putative secreted esterase	1072C>T	Arg358Stop	Pseudogene in strain 0211
2,401,441	T	C	T	SNV	CULC0102_2194 (glf)	1,197	UDP-galactopyranose mutase	80A>G	Asn27Ser	NA

*NA, not applicable; ORF, open reading frame; SNV, single-nucleotide variation.

Conventional ribotyping (Figure 1, panel A) showed that strains FH2016-1 and 0102 were closely related. However, comparison of 30 genome sequences of strains from around the world (Appendix Table 2, Figure 2) revealed that all 3 strains from Japan belong to a single phylogenetic cluster and sequence type (ST) 337. Whether the 3 isolates represent the entire population of *C. ulcerans* in Japan is unclear. However, more than half the isolates we have analyzed (»20) are ST337 (M. Iwaki and A. Yamamoto, unpub. data), suggesting a small amount of genetic diversity among the *C. ulcerans* population in Japan.

Close-up view of the phylogenetic tree showed that these strains from Japan divided into 2 different lineages. At most, 117 single nucleotide variations and 59 insertions/deletions existed between any 2 strains (Figure 2). Although this result indicated low variability among the 3 strains, it also showed that strain FH2016-1 was genetically distinct from 0102 and 0211 (Figure 2). Thus, the genome sequence analysis indicated that conventional ribotyping did not reflect lineage accurately and resulted in a misleading classification of these specimens. In contrast, MLST, which is now the preferred method of molecular typing (*8*,*10*), provided more accurate results. We queried the genomic sequences of the 3 strains on the PubMLST website (https://pubmlst.org) and analyzed them at 7 alleles (*atpA*, *dnaE*, *dnaK*, *fusA*, *leuA*, *odhA*, and *rpoB*). The same sequence type (ST337) was assigned to all 3 strains, reflecting the low genetic variability among these strains.

## Conclusions

Our study of 3 strains of *C. ulcerans* showed that conventional ribotyping is less accurate than other methods of phylogenetic analysis. In comparison, MLST is less erroneous, and whole-genome sequencing produces results with greater resolution than those of conventional ribotyping. MLST produced results with lower resolution than whole-genome sequencing while maintaining a high level of accuracy. MLST and whole-genome sequencing improve the accuracy and efficiency of phylogenetic analysis of *C. ulcerans*.

Appendix Additional information on strains, mutations, and phylogenetic analysis of *Corynebacterium ulcerans*.
