# Supplementary material for: Limitations of Ribotyping as Genotyping Method for Corynebacterium ulcerans
Source: Emerg Infect Dis. 2020 Oct;26(10):2457–9. doi: 10.3201/eid2610.200086 (PMC7510715; doi:10.3201/eid2610.200086)
Supplement: Appendix — Additional information on strains, mutations, and phylogenetic analysis of Corynebacterium ulcerans. [file 20-0086-Techapp-s1.pdf]

Article DOI: https://doi.org/10.3201/eid2610.200086

Limitations of Ribotyping as Genotyping Method for *Corynebacterium ulcerans*

Appendix

Appendix Table 1. Mutation sites in 3 strains of *Corynebacterium ulcerans*, Japan, 2001–2016*

Chromosome 0102	Position, nt	Mutation type	Strain		
			102	211	FH2016-1
AP012284.1	5468	SNV	T	T	C
AP012284.1	13074	SNV	G	G	A
AP012284.1	13243	Ins	T	TTC	T
AP012284.1	21848	SNV	G	G	A
AP012284.1	23960	SNV	C	C	T
AP012284.1	27867	Del	AGGAACTACCTCCGCGTACGCGGAGAAAAGCGTG TCTCCGGATCGAGGGAAGAACTTCTGCG	AGGAACTACCTCCGCGTACGCGGAGAAAA GCGTGTCTCCGGATCGAGGGAAGAACTTCT GCG	A
AP012284.1	28234	Del	GGGAACTACCTCCGCGTACGCGGAGAAAAGGCGT TCAGGTTTCCGCCGGCCTCAGCCAGCTT	GGGAACTACCTCCGCGTACGCGGAGAAAA GGCGTTCAGGTTTCCGCCGGCCTCAGCCA GCTT	G
AP012284.1	31398	SNV	G	G	A
AP012284.1	38660	SNV	G	G	A
AP012284.1	43805	SNV	T	T	C
AP012284.1	66246	SNV	C	C	T
AP012284.1	72966	SNV	G	G	A
AP012284.1	105558	Ins	A	A	AG
AP012284.1	109678	SNV	G	G	A
AP012284.1	147936	SNV	T	C	T
AP012284.1	148229	SNV	C	C	A
AP012284.1	157816	SNV	A	A	G
AP012284.1	219707	SNV	T	T	C
AP012284.1	270551	Del	AT	A	AT
AP012284.1	306538	Ins	C	C	CG
AP012284.1	309164	SNV	G	G	A
AP012284.1	347372	SNV	A	A	G
AP012284.1	352293	SNV	T	T	C
AP012284.1	356626	SNV	T	T	C
AP012284.1	364122	SNV	T	T	C
AP012284.1	389417	SNV	G	G	A
AP012284.1	399537	SNV	C	C	A
AP012284.1	399986	SNV	G	G	A
AP012284.1	408576	SNV	C	T	C

Chromosome 0102	Position, nt	Mutation type	Strain		
			102	211	FH2016-1
AP012284.1	448658	SNV	T	T	C
AP012284.1	471264	SNV	G	G	A
AP012284.1	471701	SNV	T	T	A
AP012284.1	484237	SNV	C	C	T
AP012284.1	502123	SNV	G	G	A
AP012284.1	520521	SNV	T	T	C
AP012284.1	528107	SNV	T	C	C
AP012284.1	531308	Ins	C	C	CCGGGTTAAAGCCGAGG
AP012284.1	531313	Ins	T	T	TTG
AP012284.1	531315	Ins	A	A	ACT
AP012284.1	531316	Ins	A	A	ACTCGTGTGGTCGCATACGGTGG
AP012284.1	531318	Ins	A	A	CCTCGCAGTGCGTGAGGTCATGGAGCCGCCAGCGAGGCTGGCAGCCCGGTTGTAG
AP012284.1	532752	SNV	T	T	C
AP012284.1	561350	Ins	A	AT	A
AP012284.1	562902	SNV	G	A	A
AP012284.1	577038	Ins	T	T	TG
AP012284.1	584561	SNV	T	T	C
AP012284.1	584754	Ins	G	G	GT
AP012284.1	599536	SNV	C	C	T
AP012284.1	601617	SNV	A	G	G
AP012284.1	604180	SNV	G	G	A
AP012284.1	611818	Del	CA	C	C
AP012284.1	619349	SNV	G	G	A
AP012284.1	626851	SNV	G	G	A
AP012284.1	634142	SNV	T	C	T
AP012284.1	648703	SNV	A	A	G
AP012284.1	692823	Del	AG	A	A
AP012284.1	734318	SNV	C	C	T
AP012284.1	739758	Ins	T	T	TTGTGCCAATAAGCGCGGTGGCGTTGGGGTAGTGATGTACGGTCTCGCGGA
AP012284.1	741335	SNV	A	A	C
AP012284.1	742304	Del	AG	AG	A
AP012284.1	770130	Ins	C	C	CGA
AP012284.1	770138	Ins	A	A	AG
AP012284.1	770142	Del	CTT	CTT	C
AP012284.1	779707	SNV	G	G	T
AP012284.1	787007	SNV	C	C	T
AP012284.1	803736	SNV	A	A	G
AP012284.1	820419	SNV	G	T	G
AP012284.1	859288	SNV	A	A	G
AP012284.1	891192	SNV	A	A	C
AP012284.1	976642	SNV	G	G	A
AP012284.1	981747	SNV	C	T	C
AP012284.1	987876	SNV	T	T	C
AP012284.1	989653	SNV	C	T	C

Chromosome 0102	Position, nt	Mutation type	Strain		
			102	211	FH2016-1
AP012284.1	991460	SNV	A	G	G
AP012284.1	1015997	Ins	C	C	CA
AP012284.1	1058136	SNV	G	A	G
AP012284.1	1072030	SNV	G	G	T
AP012284.1	1086565	SNV	G	G	A
AP012284.1	1094809	SNV	T	C	T
AP012284.1	1116335	SNV	C	T	T
AP012284.1	1140849	SNV	A	A	G
AP012284.1	1150120	SNV	G	G	T
AP012284.1	1153661	SNV	C	C	T
AP012284.1	1167108	Del	TG	TG	T
AP012284.1	1177278	Ins	A	A	AGG
AP012284.1	1187307	SNV	G	G	T
AP012284.1	1193209	SNV	G	G	A
AP012284.1	1202772	SNV	T	T	A
AP012284.1	1211380	SNV	G	G	A
AP012284.1	1221468	SNV	C	C	T
AP012284.1	1237855	SNV	T	T	C
AP012284.1	1239122	SNV	G	G	A
AP012284.1	1249696	SNV	C	C	T
AP012284.1	1256330	Ins	G	G	GT
AP012284.1	1265029	SNV	C	C	A
AP012284.1	1271793	SNV	A	A	G
AP012284.1	1275272	SNV	C	C	T
AP012284.1	1276489	SNV	T	T	A
AP012284.1	1359233	Del	ACTAACCAGAATTTTGACGGAGCCCTAAACTTCACCGGCTGCAGTACTGGGAGCTTGTCAGGAAAATTTGAGCAGCGCGAGGGTGGACTTTTCCACGATG	ACTAACCAGAATTTTGACGGAGCCCTAAACTTCACCGGCTGCAGTACTGGGAGCTTGTCAGGAAAATTTGAGCAGCGCGAGGGTGGACTTTTCCACGATG	A
AP012284.1	1359874	Del	CA	CA	C
AP012284.1	1360594	Ins	C	C	CTCATCGTCTACGACTACGCAGACCACAGAGCCAACGTCCTCATCGTCTACGACTACGCAGACCACGGAACCAACGTCGTCATCGTCTACGACTACGCAGACCACGGAACCAACGTCG
AP012284.1	1365000	Ins	T	T	TTTTTA
AP012284.1	1365003	Del	CTAAAG	CTAAAG	C
AP012284.1	1394742	Del	GCAC	GCAC	G
AP012284.1	1394745	Ins	C	C	CGT
AP012284.1	1394748	Del	TC	TC	T
AP012284.1	1394782	Del	CT	CT	C
AP012284.1	1462352	SNV	A	C	A
AP012284.1	1501754	SNV	G	A	A
AP012284.1	1511297	SNV	C	C	A
AP012284.1	1525192	SNV	G	G	A
AP012284.1	1527488	SNV	A	A	G
AP012284.1	1530546	Del	GC	G	G

Chromosome 0102	Position, nt	Mutation type	Strain		
			102	211	FH2016-1
AP012284.1	1549974	Del	CA	CA	C
AP012284.1	1615719	Ins	A	A	ACAGCAGGTTTTGGAGCGGCTGGCTTCGCGGAAGCCGGCTTGGGTGCTGCAGGCTTTGCGG
AP012284.1	1681351	SNV	A	A	G
AP012284.1	1683276	SNV	T	T	G
AP012284.1	1709596	SNV	G	T	G
AP012284.1	1725453	SNV	A	G	G
AP012284.1	1741037	Del	GA	GA	G
AP012284.1	1756915	SNV	G	G	A
AP012284.1	1768592	SNV	A	A	G
AP012284.1	1780351	SNV	C	C	T
AP012284.1	1787891	SNV	A	A	G
AP012284.1	1789178	SNV	T	T	A
AP012284.1	1817372	SNV	A	A	G
AP012284.1	1819250	SNV	C	C	T
AP012284.1	1835048	SNV	T	T	A
AP012284.1	1847653	Ins	T	T	TG
AP012284.1	1886000	SNV	C	T	C
AP012284.1	1886801	SNV	T	T	C
AP012284.1	1909561	Ins	C	C	CG
AP012284.1	1931545	SNV	C	C	T
AP012284.1	1936049	SNV	C	C	T
AP012284.1	1943886	SNV	G	G	A
AP012284.1	1963329	SNV	C	C	T
AP012284.1	1967703	SNV	T	T	C
AP012284.1	1977322	SNV	G	G	T
AP012284.1	2007494	SNV	T	C	C
AP012284.1	2025107	Del	GATA	GATA	G
AP012284.1	2034743	SNV	G	G	A
AP012284.1	2038449	Del	GGCGCGGCTTTTCTCCGCGTATGCGGAGGTAGTTCCCACCGCCGGGCAGCACTCCCTCAAAGCACAAGCTTTTCTCCGCGTATGCGGAGGTAGTTCCGGATCGGGCGCGGGCTGCGATTTCGA	GGCGCGGCTTTTCTCCGCGTATGCGGAGGTAGTTCCCACCGCCGGGCAGCACTCCCTCAAAGCACAAGCTTTTCTCCGCGTATGCGGAGGTAGTTCCGGATCGGGCGCGGGCTGCGATTTCGA	G
AP012284.1	2038758	Del	GGCCTTTTCTCCGCGTATGCGGAGGTAGTTCCCATCATTGGGGAATCCCCCCGAGCATGGGTGACTTTTCTCCGCGTATGCGGAGGTAGTTCCCATCCGGCGTTAGTGACCGCTGCGGTCGCA	G	GGCCTTTTCTCCGCGTATGCGGAGGTAGTTCCCATCATTGGGGAATCCCCCCGAGCATGGGTGACTTTTCTCCGCGTATGCGGAGGTAGTTCCCATCCGGCGTTAGTGACCGCTGCGGTCGCA
AP012284.1	2039309	Del	GGCTTTTCTCCGCGTATGCGGAGGTAGTTCCGCAAATCTTCAAGAACATCTTTGTCCAAGGTCCTTTTCTCCGCGTATGCGGAGGTAGTTCTGAGCTGGGTTTTGTGGCGTCTCTTTCTCGTC	GGCTTTTCTCCGCGTATGCGGAGGTAGTTCCGCAAATCTTCAAGAACATCTTTGTCCAAGGTCCTTTTCTCCGCGTATGCGGAGGTAGTTCTGAGCTGGGTTTTGTGGCGTCTCTTTCTCGTC	G
AP012284.1	2040619	Del	TCCAC	TCCAC	T
AP012284.1	2040630	Del	CG	CG	C

Chromosome 0102	Position, nt	Mutation type	Strain		
			102	211	FH2016-1
AP012284.1	2040634	Del	GGC	GGC	G
AP012284.1	2040639	Ins	A	A	AGCTGGTC
AP012284.1	2040645	Del	GCCCC	GCCCC	G
AP012284.1	2040650	Ins	G	G	GA
AP012284.1	2040653	Ins	G	G	GGAA
AP012284.1	2054399	Ins	T	T	TA
AP012284.1	2055051	Ins	A	A	AC
AP012284.1	2057376	SNV	G	G	T
AP012284.1	2059553	SNV	C	C	T
AP012284.1	2066689	Del	GAA	GAA	G
AP012284.1	2066698	Ins	T	T	TC
AP012284.1	2066705	Ins	C	C	CCT
AP012284.1	2084128	SNV	G	G	A
AP012284.1	2104276	SNV	G	A	G
AP012284.1	2126945	SNV	C	T	C
AP012284.1	2131950	SNV	G	G	A
AP012284.1	2147458	SNV	G	G	A
AP012284.1	2157633	SNV	G	G	T
AP012284.1	2159106	SNV	G	G	A
AP012284.1	2247082	Del	CA	CA	C
AP012284.1	2258634	SNV	C	C	T
AP012284.1	2261240	SNV	T	T	C
AP012284.1	2276928	Del	TC	T	T
AP012284.1	2276937	Del	AC	AC	A
AP012284.1	2283843	Ins	A	A	AG
AP012284.1	2284180	SNV	T	T	G
AP012284.1	2284351	SNV	G	G	A
AP012284.1	2295862	SNV	C	C	T
AP012284.1	2296083	SNV	T	T	C
AP012284.1	2299003	SNV	C	C	T
AP012284.1	2324071	SNV	T	T	C
AP012284.1	2395978	SNV	G	G	A
AP012284.1	2400888	SNV	A	C	C
AP012284.1	2401441	SNV	T	C	T
AP012284.1	2416052	SNV	C	C	T
AP012284.1	2417504	SNV	G	G	T
AP012284.1	2421828	Ins	C	C	CT
AP012284.1	2450685	SNV	C	C	G
AP012284.1	2468623	Ins	A	ACAGCGTGTCAG	ACAGCGTGTCAG
AP012284.1	2487864	SNV	C	T	T
AP012284.1	2496366	Ins	C	C	CA
AP012284.1	2515718	SNV	A	A	G
AP012284.1	2549156	SNV	T	T	G
AP012284.1	2560560	SNV	T	T	C

*Del, deletion; Ins, insertion; SNV, single-nucleotide variation. Dashes indicate not applicable.

Appendix Table 2. Strain information used in core genome phylogenetic analysis of *Corynebacterium ulcerans**

Strain	Country	Sequence type	tox (%)¶	BioSample ID	Assembly ID	Sequence completion status
PO100/5	Portugal	New ST	94.77	SAMN07109093	GCA_002162115.1	Complete
KL1196	Germany	ST578	94.66	SAMN10477295	GCA_004382105.1	Contig
W25	Germany	ST578	94.66	SAMN12027930	GCA_006370535.1	Contig
FRC11	France	ST335	–	SAMN03099770	GCA_000767685.1	Complete
NCTC8666	NA	ST335	95.19	SAMEA3923577	GCA_900447245.1	Contig
NCTC12077	NA	ST335	–	SAMN02470561	GCA_000498915.1	Contig
03-8664	France	ST341	99.23	SAMN03938368	GCA_001298285.2	Contig
131002	Brazil	New ST	–	SAMN03418694	GCA_000968945.1	Complete
04-7514	France	ST344	99.7	SAMN04041014	GCA_001302345.1	Contig
LSPQ-04227	Canada	ST344	98.57	SAMN03387456	GCA_000988285.1	Contig
LSPQ-04228	Canada	ST344	98.57	SAMN03387457	GCA_000988295.1	Contig
210932	Brazil	ST329	99.47	SAMN03025288	GCA_000767415.1	Complete
131001	Brazil	ST329	99.47	SAMN03316967	GCA_001281445.1	Complete
NCTC7908	NA	ST552	–	SAMEA3893446	GCA_900475635.1	Complete
NCTC8639	UK	ST345	–	SAMEA4017706	GCA_900475775.1	Complete
NCTC7910	UK	ST345	–	SAMEA4504038	GCA_900187135.1	Complete
2590	Brazil	New ST	–	SAMN06029387	GCA_002866525.1	Contig
4940	Belarus	ST349	–	SAMN04497012	GCA_002872395.1	Contig
809	Brazil	ST338	–	SAMN02603079	GCA_000215645.1	Complete
BR-AD 2649	Brazil	New ST	–	SAMN06029388	GCA_002866485.1	Contig
FRC58	France	ST328	99.7	SAMN02471303	GCA_000499805.2	Complete
210931	Brazil	ST332	99.7	SAMN03025287	GCA_000767645.1	Complete
05146	Brazil	ST331	99.64	SAMN03104569	GCA_000769635.1	Complete
BR-AD22	Brazil	ST339	–	SAMN02603080	GCA_000215665.1	Complete
MRi49	UK	ST543	95.19	SAMN13521090	GCA_009789155.1	Complete
04-3911	France	New ST	100.00	SAMN03938367	GCA_001298275.1	Contig
KZN-2016-48390	South Africa	ST325	100.00	SAMN05729438	GCA_001876325.1	Contig
FH2016-1	Japan	ST337	100.00	SAMD00168434	GCA_009002285.1	Complete
0102	Japan	ST337	100.00	SAMD00012381	GCA_000306825.1	Complete
0211	Japan	ST337	100.00	SAMD00168433	GCA_008995395.1	Complete

*NA, not available; ST, sequence type. Dashes indicate strains without the toxin gene.
¶ nucleotide identity % against diphtheria-like toxin of str. 0102.

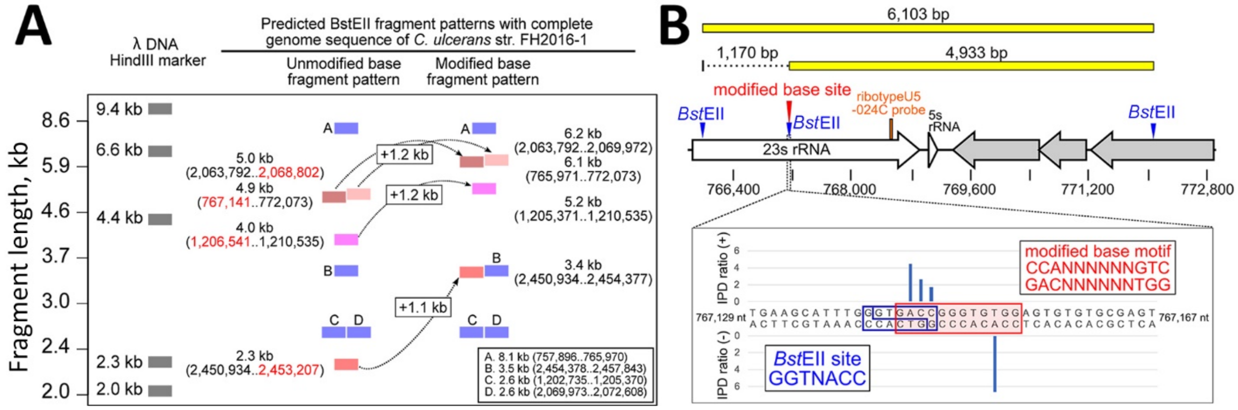

Appendix Figure 1. Alteration of ribotyping patterns by genomic DNA modification of *Corynebacterium ulcerans* strains 0102, 0211, and FH2016–1, Japan, 2001–2016. A) Fragments affected by DNA modification indicated by the pink, brown, magenta, and orange horizontal bars. Unaffected fragments (A, B, C, and D) indicated by blue horizontal bars; their sizes are displayed in the square at the lower right corner of the panel. B) Inhibition of *Bst*EII digestion. The genomic region around nt 770,000 is shown. The inset indicates the *Bst*EII site affected by genomic DNA modification that contributes to the shift in fragment size and mobility. The staggered blue frame represents the *Bst*EII site. The base motif (red rectangle) and target nucleotides for modification (blue bars), were identified by the PacBio modification analysis (Pacific Biosciences of California, Inc., https://www.pacb.com) using a motif finding algorithm (*1*). Longer bars represent higher relative probability of modification.

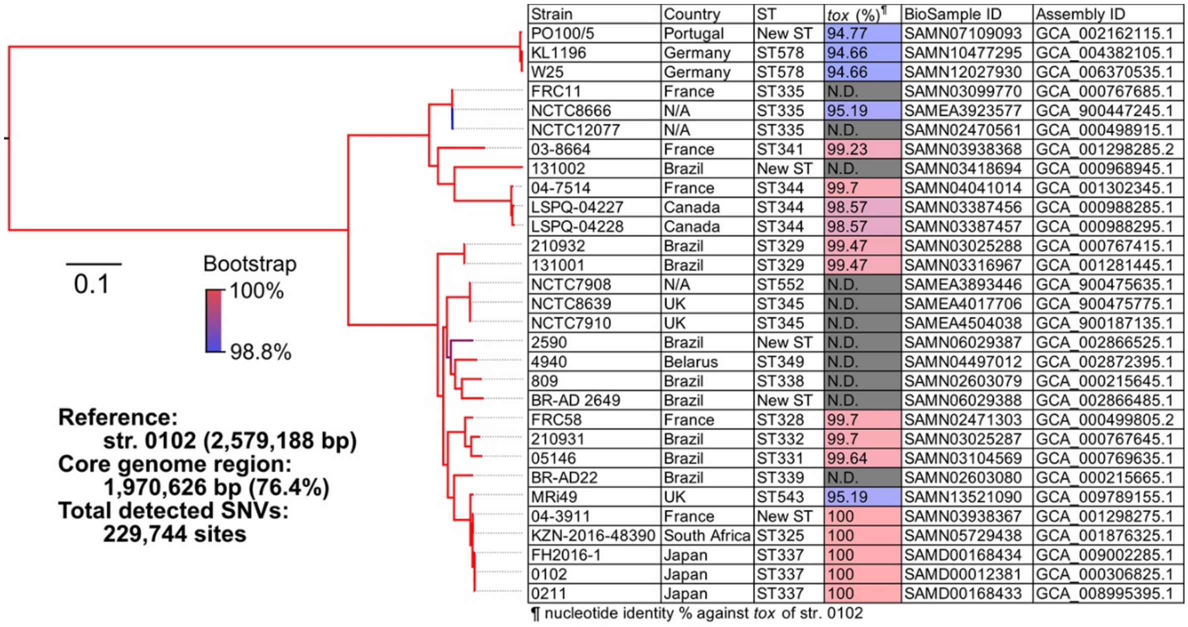

Strain	Country	ST	*tox* (%)¶	BioSample ID	Assembly ID
PO100/5	Portugal	New ST	94.77	SAMN07109093	GCA_002162115.1
KL1196	Germany	ST578	94.66	SAMN10477295	GCA_004382105.1
W25	Germany	ST578	94.66	SAMN12027930	GCA_006370535.1
FRC11	France	ST335	N.D.	SAMN03099770	GCA_000767685.1
NCTC8666	N/A	ST335	95.19	SAMEA3923577	GCA_900447245.1
NCTC12077	N/A	ST335	N.D.	SAMN02470561	GCA_000498915.1
03-8664	France	ST341	99.23	SAMN03938368	GCA_001298285.2
131002	Brazil	New ST	N.D.	SAMN03418694	GCA_000968945.1
04-7514	France	ST344	99.7	SAMN04041014	GCA_001302345.1
LSPQ-04227	Canada	ST344	98.57	SAMN03387456	GCA_000988285.1
LSPQ-04228	Canada	ST344	98.57	SAMN03387457	GCA_000988295.1
210932	Brazil	ST329	99.47	SAMN03025288	GCA_000767415.1
131001	Brazil	ST329	99.47	SAMN03316967	GCA_001281445.1
NCTC7908	N/A	ST552	N.D.	SAMEA3893446	GCA_900475635.1
NCTC8639	UK	ST345	N.D.	SAMEA4017706	GCA_900475775.1
NCTC7910	UK	ST345	N.D.	SAMEA4504038	GCA_900187135.1
2590	Brazil	New ST	N.D.	SAMN06029387	GCA_002866525.1
4940	Belarus	ST349	N.D.	SAMN04497012	GCA_002872395.1
809	Brazil	ST338	N.D.	SAMN02603079	GCA_000215645.1
BR-AD 2649	Brazil	New ST	N.D.	SAMN06029388	GCA_002866485.1
FRC58	France	ST328	99.7	SAMN02471303	GCA_000499805.2
210931	Brazil	ST332	99.7	SAMN03025287	GCA_000767645.1
05146	Brazil	ST331	99.64	SAMN03104569	GCA_000769635.1
BR-AD22	Brazil	ST339	N.D.	SAMN02603080	GCA_000215665.1
MRi49	UK	ST543	95.19	SAMN13521090	GCA_009789155.1
04-3911	France	New ST	100	SAMN03938367	GCA_001298275.1
KZN-2016-48390	South Africa	ST325	100	SAMN05729438	GCA_001876325.1
FH2016-1	Japan	ST337	100	SAMD00168434	GCA_009002285.1
0102	Japan	ST337	100	SAMD00012381	GCA_000306825.1
0211	Japan	ST337	100	SAMD00168433	GCA_008995395.1

¶ nucleotide identity % against *tox* of str. 0102

Appendix Figure 2. Phylogenetic analysis of 30 strains of *Corynebacterium ulcerans*, including 3 sequences (FH2016-1, 0102 and 0211) from Japan. Phylogenetic tree constructed with maximum-likelihood analysis. ¶nucleotide identity % against diphtheria-like toxin of str. 0102. NA, not available; ND, no data (indicates strains without toxin gene); SNV, single-nucleotide variation; ST, sequence type; str, strain.

Reference

1. Kelleher P, Murphy J, Mahony J, van Sinderen D. Identification of DNA base modifications by means of Pacific Biosciences RS sequencing technology. Methods Mol Biol. 2018;1681:127–37. PubMed https://doi.org/10.1007/978-1-4939-7343-9_10
